# Identification of Circular RNA hsa_Circ_0003391 in Peripheral Blood Is Potentially Associated With Alzheimer's Disease

**DOI:** 10.3389/fnagi.2020.601965

**Published:** 2020-12-18

**Authors:** Li Liu, Xi Chen, Yu-Hua Chen, Ke Zhang

**Affiliations:** ^1^Department of Neurology, Shenyang Fifth People Hospital, Shenyang, China; ^2^Department of Developmental Cell Biology, Key Laboratory of Cell Biology, Ministry of Public Health, Key Laboratory of Medical Cell Biology, Ministry of Education, China Medical University, Shenyang, China

**Keywords:** circular RNA, hsa_circ_0003391, Alzheimer's disease, peripheral blood, non-coding RNA

## Abstract

Circular RNAs (circRNAs) have recently been discovered as a novel type of endogenous non-coding RNA that may regulate gene expression in mammals. In the central nervous system (CNS), circRNAs are relevant to many neurological disorders such as Alzheimer's disease (AD). In this study, we attempted to identify an aberrant circRNA, hsa_circ_0003391, which is significantly downregulated in the peripheral blood of patients with AD, and to explore the relationship between hsa_circ_0003391 and the clinical manifestation of AD. The expression of hsa_circ_0003391 had a specific decrease in the peripheral blood of patients with AD compared to those with other types of dementia. To evaluate the potential diagnostic value of the circRNA, we performed a receiver operating characteristic (ROC) curve analysis. The area under the curve (AUC) value was 0.7283 for hsa_circ_0003391, which was statistically significant. The natural form of hsa_circ_0003391 in the peripheral blood was a loop structure with good stability. We found a potential correlation between the expression of hsa_circ_0003391 and the clinical manifestations of AD. Bioinformatic analysis was carried out to predict the latent target microRNAs (miRNA) of hsa_circ_0003391. Furthermore, microRNAs targeted by hsa_circ_0003391 were successfully detected, and miR-574-5p had an expected elevation in the AD groups, suggesting that miR-574-5p might be a potential microRNA target for hsa_circ_0003391. Our results suggest that the downregulation of hsa_circ_0003391 in the peripheral blood has a potential relationship with AD. Our findings not only provide an important latent biomarker but also highlight an important perspective for the following study into AD pathogenesis. This may promote the process of novel therapeutics targeting non-coding RNA.

## Introduction

Circular RNAs (circRNAs) have lately been discovered and described as a novel type of endogenous non-coding RNAs that may regulate mammalian gene expression. The main characteristic of circRNAs is a covalent closed loop structure, which has neither 5′ to 3′ polarity nor a polyadenylated tail (Chen and Yang, 2015). CircRNA transcripts were previously thought to be only rarely expressed (Jeck et al., 2013; Memczak et al., 2013; Chen and Yang, 2015). However, recently, many circRNAs have been identified in both humans and mice by two independent studies (Jeck et al., 2013; Memczak et al., 2013). Numerous circRNAs in mammalian cells are conservative and steady, appearing specifically expressed in certain cells or at their developmental stages (Zhou and Yang, 2012; Memczak et al., 2013), which indicate that circRNAs are of vital importance in many physiological and pathological processes. Natural endogenous circRNAs contain selectively conserved microRNA (miRNA) target sites, which are presumed to function as miRNA “sponges” in mammalian cells. Accumulating research displays that circRNAs are crucial in gene expression regulation by restraining the miRNA activity, which is involved in almost all cellular activities, such as cell proliferation, movement, differentiation, and death (Zhou and Yang, 2012).

Recently, some circRNAs originate from genomic loci that are relevant to AD, suggesting that circRNAs may regulate specific processes of pathophysiology (Huang et al., 2015; Li et al., 2015; Han et al., 2017). In the central nervous system (CNS), circRNAs are relevant to some neurological disorder, for example, Alzheimer's disease (AD), Parkinson's disease, multiple sclerosis, and schizophrenia (Ghosal et al., 2013; Lukiw, 2013). With the ability of small molecules to pass through the blood–brain barrier (BBB) and enter the blood, when circRNAs are combined with other biomarkers and imaging tools, diagnostic capabilities can be enhanced effectively. In addition, circRNAs are rich in the brain and within exosomes. The capability of these circRNAs to cross the BBB makes them the ideal potential diagnostic tools for CNS disorders (Lu and Xu, 2016).

Alzheimer's disease (AD) is one of the most common age-related dementia (accounting for 60%), in all confirmed cases of dementia (Honjo et al., 2012). AD is characterized by progressive degeneration of the CNS, which ultimately leads to loss of cognitive function. Most AD diagnoses are based on clinical findings, β-amyloid and phosphorylated tau protein measurements in the cerebrospinal fluid, and a number of relevant neuroimaging methods (Sperling et al., 2014; Lan et al., 2015). However, a lumbar puncture is a fairly invasive procedure and can be challenging to perform in elderly infirm individuals (Sperling et al., 2014; Lan et al., 2015). Moreover, neuroimaging methods such as MRI of specific brain regions (i.e., the hippocampus), amyloid tracer imaging, and functional MRI require special equipment with high cost (Maruyama et al., 2013; Sperling et al., 2014; Lan et al., 2015). In consequence, the analysis of biological markers obtained from the cerebrospinal fluid and neuroimaging has significant limitations in their clinical application for the diagnosis of AD. Exploring the biomarkers in peripheral blood of patients with AD, combined with clinical symptoms, may assist in formulating a diagnosis of AD. Furthermore, the downstream miRNAs of circRNAs have multifaceted functions in promoting or restricting the process of AD. For example, microRNA-455-3p is not only regarded as a potential peripheral biomarker for AD (Kumar et al., 2017; Kumar and Reddy, 2020) but also can modulate the amyloid protein precursor processing and enhance mitochondrial biogenesis and synaptic activity (Kumar et al., 2017, 2019; Kumar and Reddy, 2019, 2020). Therefore, investigating the abnormal circRNAs and their downstream miRNAs in patients with AD will contribute to the exploration of the molecular mechanism of AD pathogenesis.

In the present study, we identified a circRNA hsa_circ_0003391 originating from ubiquitin-associated and SH3 domain-containing protein B (UBASH3B), which was significantly downregulated in the peripheral blood of patients with AD and closely related to the clinical manifestation of AD. Our results indicate that hsa_circ_0003391 may be of vital importance in AD pathogenesis.

## Materials and Methods

### Patients

In this study, 50 Chinese patients with AD, 50 age-matched control subjects, 20 patients with dementia with Lewy bodies (DLB), and 40 patients with vascular dementia (VD) were recruited by the Shenyang Fifth People's Hospital and the First Affiliated Hospital of China Medical University (Table 1). According to the National Institute of Neurological and Communication Disorders and the Stroke-and-Alzheimer's Disease and Related Disorders Association (NINCDS-ADRDA), the diagnostic criteria for possible AD was applied using clinical neurologic examination, neuropsychological assessment, electroencephalogram, and MRI or CT. Routine laboratory tests were performed to exclude the other dementia (McKhann et al., 1984, 2011; Liu et al., 2015). The control subjects were chosen according to the following criteria: no complaints of memory impairment (Liu et al., 2015); general cognitive performance informed by a reliable evaluator (Liu et al., 2015); normal Mini-Mental State Examination (MMSE) (Folstein et al., 1975; Liu et al., 2015) score according to their age, sex, and educational background; no family history of dementia; and no obvious comorbidities that could interfere with the brain function (Liu et al., 2015). DLB was diagnosed according to the 2005 and 2017 McKeith consensus criteria (McKeith et al., 2017). The diagnostic criteria of the National Institute of Neurological Disorders and Stroke and Association Internationale pour la Recherché et l'Enseignement en Neurosciences (NINDS-AIREN) were used to define VD (Román et al., 1993). All subjects underwent psychological evaluations, including the MMSE, Montreal Cognitive Assessment (MoCA) (Nasreddine et al., 2005), and the Clinical Dementia Rating (CDR) (Morris, 1993), to assess the cognitive function and the severity of impairment. In addition, the Rey Auditory Verbal Learning Test (RAVLT) was administered to assess the item memory. The RAVLT started with a list of 15 words which an examiner read aloud at a rate of one word per second. For the RAVLT immediate recall (RAVLT-I) analysis, the participant was asked to immediately recall as many words as they could remember. After 30 min, RAVLT delayed recall (RAVLT-D) was assessed using interpolated testing, in which the participant was asked to make a delayed recall of the list of words again. This study was approved by the local ethics committee (Liu et al., 2015), and the written informed consents, including introduction of the project, research purpose, research process, potential benefits and risks, the right to participate, and confidentiality, were obtained from all the participants.

**Table 1:** Characteristics of the study participants.

**Characteristics**	**Control**	**AD**	**VD**	**DLB**
Enrollment time	12 months	12 months	12 months	12 months
Total number	50	50	40	20
Age (mean ± SD)	68.17 ± 2.74	68.23 ± 1.95	69.6 ± 1.89	67.4 ± 1.44
Sex (Male/Female)	25/25	26/24	20/20	11/9
Education (mean ± SD)	11.6 ± 0.58	10.9 ± 0.84	10 ± 1.25	9.8 ± 1.11
MMSE (mean ± SD)	29.83 ± 0.42	16.24 ± 2.11	15.23 ± 1.76	16.63 ± 1.45
MoCA (mean ± SD)	26.23 ± 0.41	10.86 ± 1.08	11.4 ± 1.29	11.1 ± 1.28
RAVLT: immediate recall (mean ± SD)	29.87 ± 0.28	7.98 ± 0.94	12.1 ± 2.43	11.85 ± 1.22
RAVLT: delayed recall (mean ± SD)	12.13 ± 0.44	0.94 ± 0.33	3.25 ± 0.11	2.32 ± 0.63
CDR (mean ± SD)	0 ± 0	7.27 ± 0.23	8.45 ± 0.18	7.25 ± 0.65
Left hippocampal volume (mean± SD)	2,684.84 ± 617.22	1,989.74 ± 536.98	2,721.74 ± 641.65	2,677.55 ± 536.64
Right hippocampal volume (mean± SD)	2,663.61 ± 610.75	1,986.25 ± 521.46	2,691.06 ± 612.53	2,653.74 ± 567.71
Total hippocampal volume (mean± SD)	5,348.45 ± 1,225.79	3,975.99 ± 1,046.26	5,412.8 ± 1,249.86	5,331.29 ± 1,126.89

### Prepare Clinical Specimens

Approximately 10 ml of the peripheral whole blood was sampled from each subject by venipuncture and converged into test tubes containing dipotassium ethylenediaminetetraacetic acid. The plasma and the whole blood cells were separated by centrifugation at 800 *g* for 10 min. During the collection of clinical samples, the peripheral blood samples were stored at varying temperatures. To identify the stability of circRNAs at different temperatures, the peripheral blood samples from five patients with AD were randomly chosen, and each sample was divided into four equal parts. Then, the samples were stored at three separate temperatures (25, 4, and −20°C) for 0, 2, 6, and 12 h. The RNA of the plasma or the whole blood cells was extracted according to the manufacturer's instructions.

### Microarray Analysis

Total RNA from fresh blood plasma supernatant was isolated using a TRizol reagent. The total RNA was digested with Ribonuclease R to remove linear RNAs and enrich the circRNAs. The concentrations of the RNA were then determined by OD260 using a NanoDrop instrument, and the integrity was assessed by agarose gel electrophoresis. The circRNAs were then amplified and transcribed into fluorescent circRNAs (Arraystar Super RNA Labeling Kit; Arraystar). The purified, labeled circRNAs were performed using the microarray analysis according to the manufacturer's instructions. When comparing two groups with different profiles, the “fold change,” an absolute fold change between the two groups, for each circRNA was calculated. The statistical significance of the expression difference was estimated by using Student's *t*-test. CircRNAs with absolute fold changes ≥1.5 and values of *p* < 0.05 were determined to be differentially expressed.

### Real-Time Polymerase Chain Reaction

A quantitative real-time PCR (RT-qPCR) was performed using an ABI 7500 RT-PCR system with a SYBR Premix Ex Taq Kit. Glyceraldehyde-3-phosphate dehydrogenase (GAPDH) was selected as the reference gene (Li et al., 2018). The amplification conditions for the RT-qPCR were as follows: 95°C for 10 s, followed by 40 cycles at 95°C for 5 s and 60°C for 32 s. The standard curves method was used to determine the amount of hsa_circ_0003391 transcripts of individual samples. A recombinant pMD 18-T vector containing the target circRNA or reference gene cDNA was constructed by plasmid cloning, which was used to make the standard curves. The copy numbers of the targets were obtained from the standard curve. The copy number of target circRNAs of the individual samples was normalized to the reference gene. For analyzing relative change in the gene expression, a 2^−ΔΔ*CT*^ method was used. The amplified products were verified by DNA sequencing.

### Reverse Transcription Polymerase Chain Reaction

The total RNA from the peripheral blood was reverse transcribed by using Moloney murine leukemia virus reverse transcriptase. The RT-PCR was carried out on an ABI 7500 PCR system. The divergent primer sequences for hsa_circ_0003391 were GATCTGCGGGAAGAACAA (forward) and AACCAGTCACATGCTGCC (reverse). The amplification conditions were as follows: 30 cycles of 94°C for 5 s, 56°C for 20 s, and 72°C for 30 s. The PCR products were verified by DNA sequencing.

### Prediction of miRNAs for hsa_Circ_0003391 and Stem-Loop RT-qPCR Assay

To determine the potential miRNAs of hsa_circ_0003391, the five highest-ranking binding miRNAs (Top-5) were identified by Arraystar's homemade prediction software for the miRNA target based on TargetScan (Enright et al., 2003) and miRanda (Pasquinelli, 2012). The miRNAs were 17–24 nucleotides (nt) in length. The RT-qPCR methods required a template which was at least twice the length of either of the specific forward or reverse primers. Therefore, the stem-loop was chosen as the RT-qPCR method for identification and qualification of miRNAs. According to this method, we designed the primers for amplification by PCR, and RNU6B (U6) was used as a reference gene for miRNAs (Table 2). For reverse transcription, strand cDNA synthesis with a highly stable stem-loop primer was performed to lengthen the miRNAs from its original 20 to 60 nt. Amplification was carried out using a RT-qPCR system; for targeted miRNAs of hsa_circ_0003391, the melt curve and amplification plot were obtained.

**Table 2:** Primers for miRNAs targeted by hsa_circ_0003391.

**miRNA ID**	**Primer**	**Sequence (5^**′**^-3^**′**^)**
hsa-miR-6721-5p	RT primer	GTCGTATCCAGTGCAGGGTCCGAGGTATTCGCACTGGATACGACCTCCTAC
	Forward primer	CGGGCTGGGCAGGGGCTTATT
	Reverse primer	CGCAGGGTCCGAGGTATTC
hsa-miR-6732-5p	RT primer	GTCGTATCCAGTGCAGGGTCCGAGGT ATTCGCACTGGATACGACGGCCAGC
	Forward primer	CGGGCTAGGGGGTGGCA
	Reverse primer	CGCAGGGTCCGAGGTATTC
hsa-miR-6831-5p	RT primer	GTCGTATCCAGTGCAGGGTCCGAGGTATTCGCACTGGATACGACGACCTCC
	Forward primer	CGGGCTAGGTAGAGTGTGAGGA
	Reverse primer	CGCAGGGTCCGAGGTATTC
hsa-miR-4763-3p	RT primer	GTCGTATCCAGTGCAGGGTCCGAGGTATTCGCACTGGATACGACCCCGCCC
	Forward primer	CGGGCAGGCAGGGGCT
	Reverse primer	CGCAGGGTCCGAGGTATTC
hsa-miR-574-5p	RT primer	GTCGTATCCAGTGCAGGGTCCGAGGTATTCGCACTGGATACGACACACACT
	Forward primer	CGGGCTGAGTGTGTGTGTGT
	Reverse primer	CGCAGGGTCCGAGGTATTC
U6	RT primer	CGCTTCACGAATTTGCGTGTCAT
	Forward primer	GCTTCGGCAGCACATATACTAAAAT
	Reverse primer	CGCTTCACGAATTTGCGTGTCAT

### Statistical Analysis

Data were presented as mean ± standard deviation (SD). One-way ANOVA and *post-hoc* Holm–Sidak method were used for statistical analyses of data. Student's *t*-test was used for direct comparisons between two groups. A receiver operating characteristic (ROC) curve was generated to evaluate the diagnostic value of the circRNAs. Correlations were analyzed using Spearman's correlation coefficient. All data were analyzed by GraphPad Prism 8. A probability value of *p* < 0.05 was considered statistically significant.

## Results

### Decreased Expression of hsa_Circ_0003391 in the Plasma and the Whole Blood Cells of Patients With AD

**Figure 1:**
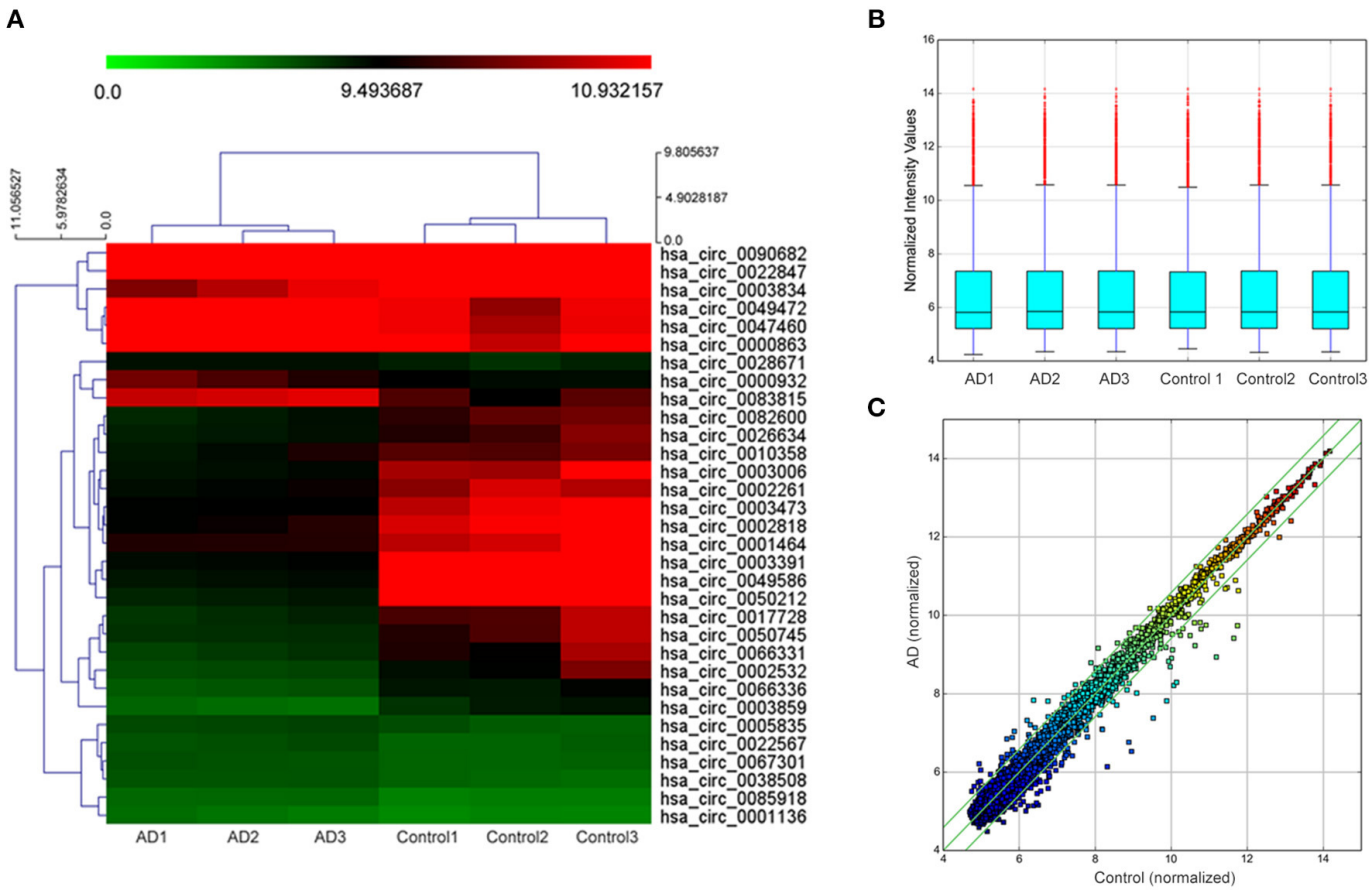
Hierarchical clustering dendrogram and heat map. **(A)** Hierarchical clustering based on the expression value of circularRNA (circRNA) shows a distinguishable gene expression profile among the samples. The green strip indicates low relative expression, and the red strip indicates high relative expression. **(B)** Box plots show the distribution of circRNAs in the two groups, which were similar after normalization. **(C)** Scatter plot indicates a change of >1.5-fold in circRNA levels between the two groups.

**Figure 2:**
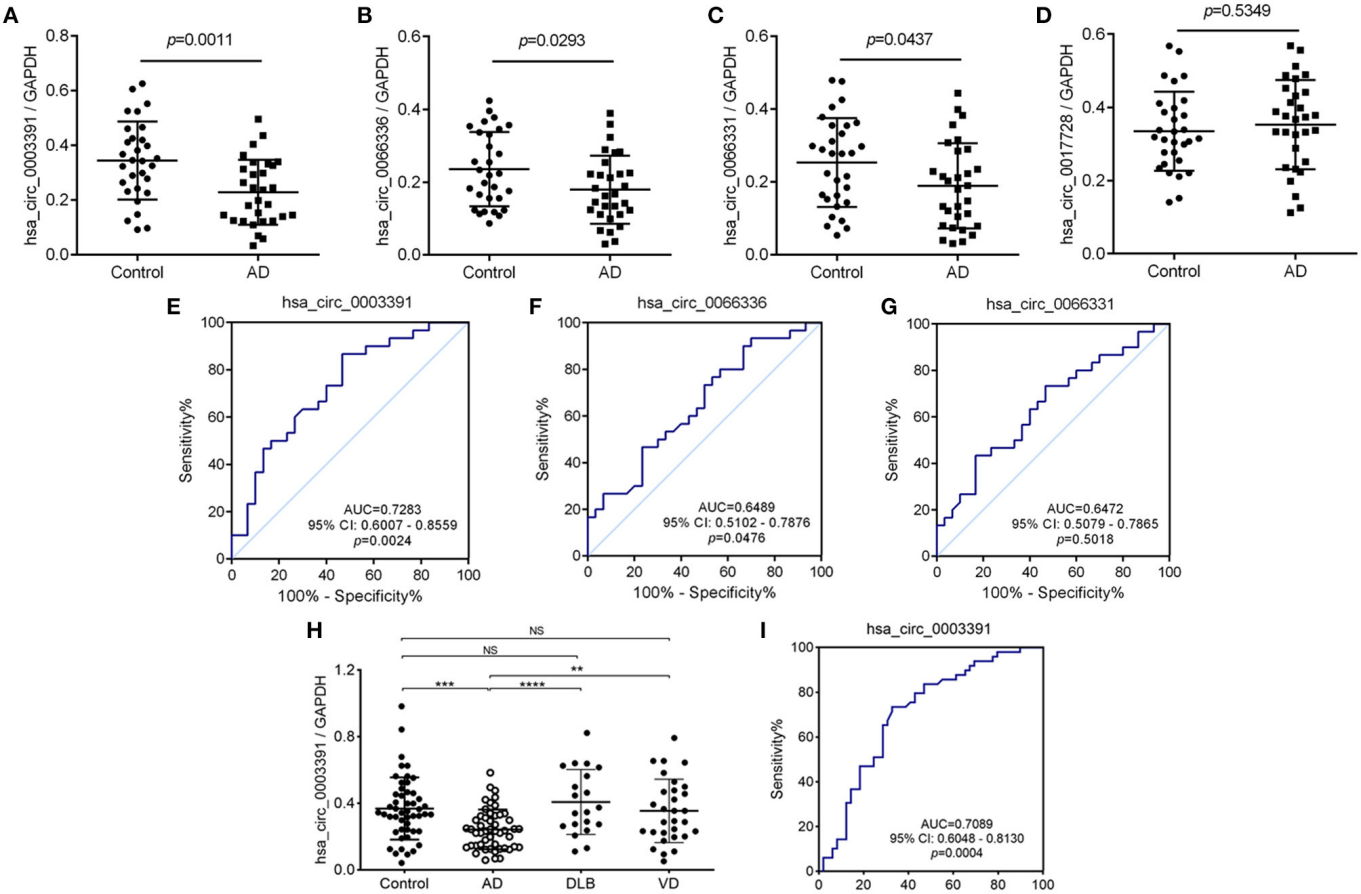
Deregulated hsa_circ_0003391 in the peripheral blood of patients with AD compared to patients with other types of dementia. The expression of hsa_circ_0003391 from the plasma of 30 patients with Alzheimer's disease (AD) and 30 age-matched controls by quantitative real-time PCR (RT-Qpcr) **(A–D)**. The receiver operating characteristic (ROC) curve analysis of hsa_circ_0003391 in the AD group is shown and the area under the curve (AUC) values are provided on the graphs **(E–G)**. RT-qPCR validation assay of the expression levels of hsa_circ_0003391 in the peripheral blood cells of 50 patients with AD, 50 age-matched controls, 20 patients with dementia with Lewy bodies (DLB), and 40 patients with vascular dementia (VD) **(H)**. The ROC curve analysis of the expression of hsa_circ_0003391 in AD blood cells is shown **(I)**. Data were analyzed using Student's *t*-test and a one-way ANOVA. **p < 0.01, ***p < 0.001, ****p < 0.0001.

In the initial stage, we collected the plasma from patients with AD to perform a circRNA microarray analysis to preliminarily screen the aberrant circRNAs in patients with AD (Figures 1A–C). In the validation stage, the RT-qPCR analysis was used to validate several candidate circRNAs, including hsa_circ_0050212 (4.74-fold), hsa_circ_0049586 (4.69-fold), hsa_circ_0003859 (4.52-fold), hsa_circ_0066336 (4.35-fold), hsa_circ_0066331 (4.17-fold), hsa_circ_0003391 (4.01-fold), hsa_circ_0050745 (3.61-fold), and hsa_circ_0017728 (3.59-fold), all of which had significant changes of >3-fold (Table 3). The results showed that except for hsa_circ_0017728, the expression of hsa_circ_0003391, hsa_circ_0066336, and hsa_circ_0066331 were obviously decreased in the plasma of patients with AD compared to the age-matched controls (Figures 2A–D). The other circRNAs, hsa_circ_0050212, hsa_circ_0049586, hsa_circ_0003859, and hsa_circ_0050745, were not detected in the samples. In addition, we also analyzed a relative change in the gene expression by using a 2^−ΔΔ*CT*^ method. We also obtained a similar variational tendency of these circRNAs (Supplementary Figure 1). To assess the diagnostic value of the circRNAs, we performed the ROC curve analysis. The area under the curve (AUC) values were 0.7283 for hsa_circ_0003391 (*p* < 0.01), 0.6489 for hsa_circ_0066336 (*p* < 0.05), and 0.6472 for hsa_circ_0066331 (*p* > 0.05; Figures 2E–G). In addition, to avoid overfitting, we did a k-fold cross-validation on these circRNAs. The accuracy for hsa_circ_0003391 was 0.6500, hsa_circ_0066331 was 0.6167, and hsa_circ_0066336 was 0.5833 (Table 4). As hsa_circ_0066336 and hsa_circ_0066331 were from the same gene, FLNB, and the ROC curve of the hsa_circ_0066331 had no statistical significance (*p* > 0.05), we selected hsa_circ_0003391 as a target circRNA to perform further study. Although the aberrant circRNAs in the plasma could be used as a potential biomarker, we focused on investigating the potential biological function of hsa_circ_0003391 in peripheral blood cells, which are the main components of the innate immunity system and are related to AD. Next, we examined the expression of hsa_circ_0003391 in the whole blood cells, from which the plasma had been removed, with an expanded sample size (*n* = 50; Table 1). The results showed that the levels of hsa_circ_0003391 in the whole blood cells of patients with AD were significantly lower than that of age-matched controls (*p* < 0.001; Figure 2H). Furthermore, we detected the specificity of hsa_circ_0003391 in the whole blood cells of patients with AD. The quantity of hsa_circ_0003391 was markedly decreased in the AD group compared to that in both the DLB and the VD groups; however, the DLB and VD groups showed similar hsa_circ_0003391 levels (Figure 2H). In order to further assess the potential of hsa_circ_0003391 as a candidate biomarker of AD, we performed the ROC curve analysis of the expression of hsa_circ_0003391 in the whole blood cells of patients with AD. The AUC values for hsa_circ_0003391 reached 0.7089, which was statistically significant (Figure 2I). These results demonstrated that the expression of hsa_circ_0003391 was downregulated in both the plasma and the whole blood cells of patients with AD. This means that the expression of circRNA hsa_circ_0003391 is downregulated in the peripheral whole blood of patients with AD.

**Table 3:** The top 10 upregulated and downregulated circRNAs detected by microarray analysis in the AD group.

**Circ ID**	**Gene Symbol**	**Location**	**Type**	**FC (abs)**	***P-*value**
**Downregulated circRNAs**					
hsa_circ_0050212	RFXANK	chr19	Exonic	4.7437	0.0191
hsa_circ_0049586	TNPO2	chr19	Exonic	4.6861	0.0201
hsa_circ_0003859	LTBP4	chr19	Exonic	4.5214	0.0461
hsa_circ_0066336	FLNB	chr3	Exonic	4.3493	0.0167
hsa_circ_0066331	FLNB	chr3	Exonic	4.1721	0.0218
hsa_circ_0003391	UBASH3B	chr11	Exonic	4.0117	0.0215
hsa_circ_0050745	CAPNS1	chr19	Exonic	3.6122	0.0211
hsa_circ_0017728	DHTKD1	chr10	Exonic	3.5956	0.0211
hsa_circ_0003006	FLNB	chr3	Exonic	2.6322	0.0147
hsa_circ_0082600	TRIM24	chr7	exonic	2.4288	0.0217
**Upregulated circRNAs**					
hsa_circ_0083815	INTS9	chr8	Exonic	1.7443	0.0119
hsa_circ_0000863	PRSS57	chr19	Sense overlapping	1.7377	0.0131
hsa_circ_0047460	FHOD3	chr18	Exonic	1.6985	0.0157
hsa_circ_0090682	HUWE1	chrX	Exonic	1.6755	0.0085
hsa_circ_0049472	PRKCSH	chr19	Exonic	1.6739	0.0296
hsa_circ_0028671	TAOK3	chr12	Exonic	1.6709	0.0284
hsa_circ_0000932	SUPT5H	chr19	Exonic	1.5994	0.0252
hsa_circ_0001136	ASXL1	chr20	Exonic	1.5949	0.0198
hsa_circ_0085918	PLEC	chr8	Exonic	1.5861	0.0318
hsa_circ_0005835	C16orf62	chr16	Exonic	1.5629	0.0443

**Table 4:** Accuracy of the three confirmed circRNAs after k-fold cross validation.

**circRNA**	**Accuracy**
hsa_circ_0003391	0.6500
hsa_circ_0066331	0.6167
hsa_circ_0066336	0.5833

### Identification of the Expression of hsa_Circ_0003391 in the Peripheral Whole Blood of Patients With AD

**Figure 3:**
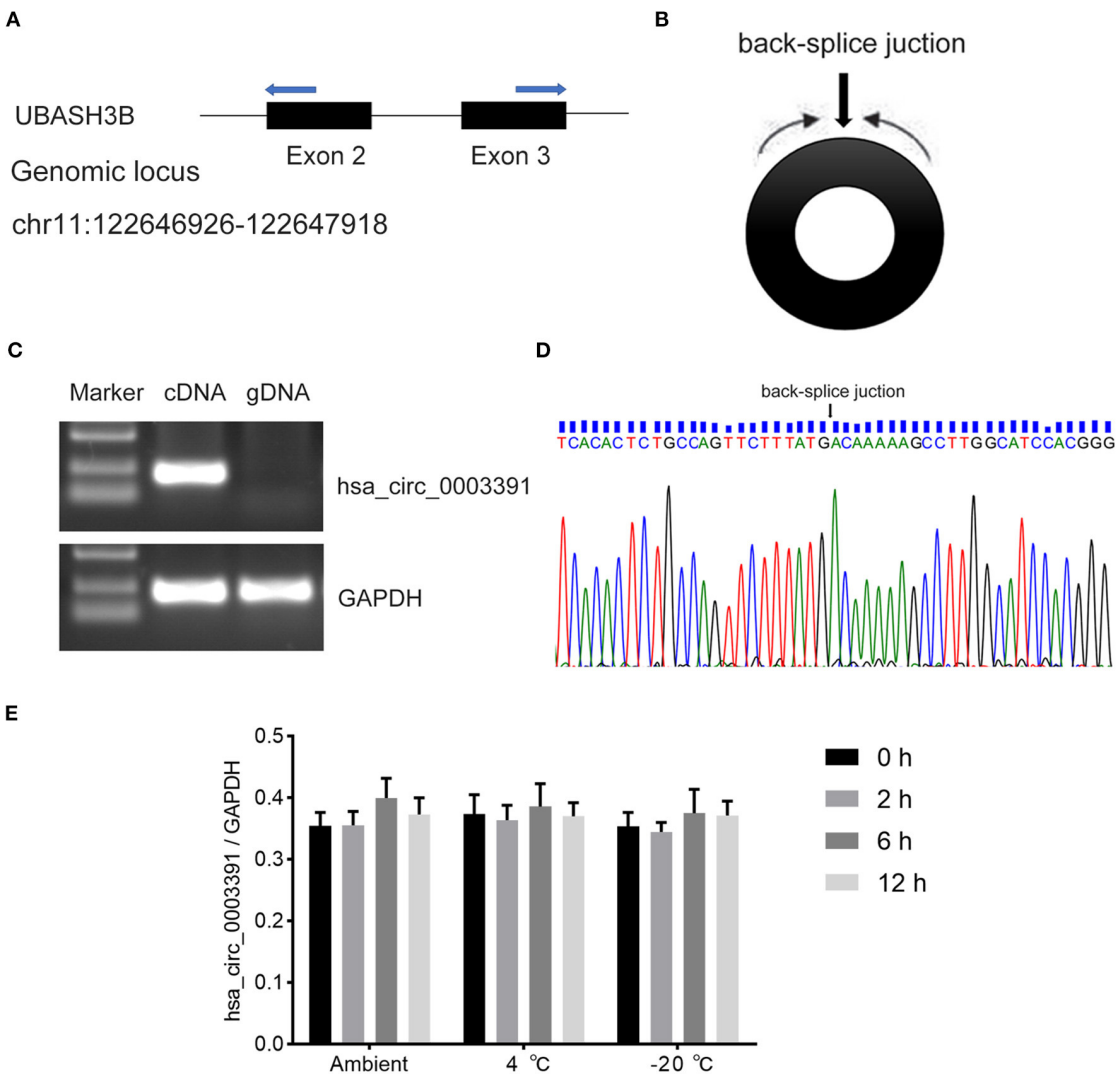
RT-PCR validation assay of hsa_circ_0003391 expression in peripheral blood of AD patients. Genomic locus of hsa_circ_0003391 **(A)**. RT-PCR products with divergent primers **(B)** of hsa_circ_0003391 verified that hsa_circ_0003391 naturally exists in the peripheral blood of patients with AD **(C)**. Sanger sequencing of hsa_circ_0003391 shows the backsplice junction **(D)**. RT-qPCR was used to detect the stability of hsa_circ_0003391 in five whole blood cell samples from patients with AD at three different temperatures (25, 4, and −20°C) for 0, 2, 6, and 12 h **(E)**.

Hsa_circ_0003391 originates from exon 2–3 of the *UBASH3B* gene locus (Figure 3A and Supplementary Figure 2). The expression of linear mRNA is produced by the genomic order of exons, which can be detected in both cDNA and genomic DNA (gDNA) by PCR primers. The backsplice junction sequence of the correctly identified circRNA should not exist in the gDNA. Therefore, the circRNA should be amplified only in the cDNA rather than in the gDNA through the circRNA-specific primers. The RT-PCR results performed with divergent primers (Figure 3B), which were used to detect the backsplice junction of the circRNA, indicated that hsa_circ_0003391 was expressed in the cDNA samples of the peripheral whole blood rather than in the gDNA (Figure 3C). The sequencing result of the RT-PCR product of hsa_circ_0003391 displayed that the backsplice junction sequence was as per that of the CircBase. The back-splice junction of hsa_circ_0003391 was confirmed by Sanger sequencing (Figure 3D), demonstrating its natural existence as a loop structure in the peripheral whole blood. In addition, these time points represented the transport and storage times for the blood samples in the study; therefore, no significant differences can be seen among samples as a result of the various time points (Figure 3E). These results suggested that hsa_circ_0003391 is present in the peripheral whole blood as a loop structure with good stability.

### Decreased hsa_Circ_0003391 Expression Correlated With the Clinical Manifestation of AD

**Figure 4:**
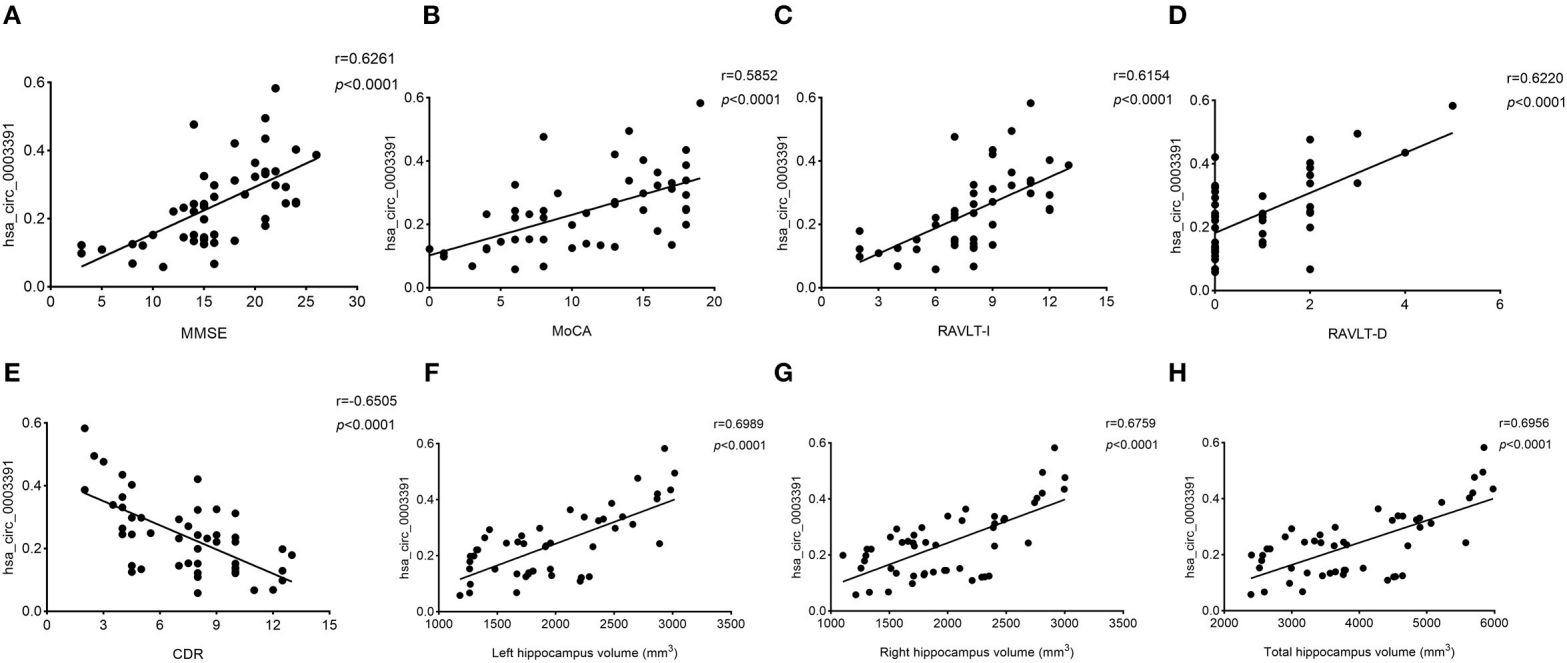
Correlations between the levels of peripheral blood hsa_circ_0003391 and the clinical examination of patients with dementia. The scatter plots showed significantly positive correlations between hsa_circ_0003391 levels in the peripheral blood and Mini-Mental State Examination (MMSE) scores **(A)**, Montreal Cognitive Assessment (MoCA) scores **(B)**, the Rey Auditory Verbal Learning Test immediate recall (RAVLT-I) scores **(C)**, and RAVLT delayed recall (RAVLT-D) scores **(D)**; significantly negative correlations between the hsa_circ_0003391 levels and the Clinical Dementia Rating (CDR) scores **(E)**; and positive correlations between hsa_circ_0003391 levels and the hippocampus volumes **(F–H)**.

Next, we explored the relationship between the hsa_circ_0003391 expression and the clinical manifestation of AD. MMSE and MoCA are the AD screening tools of choice because they can completely, accurately, and rapidly reflect a patient's intellectual status and severity of cognitive impairment. RAVLT was used to assess item memory in patients of AD, and AD severity was assessed using the CDR scale. We analyzed correlations between the expression of hsa_circ_0003391 and AD; the level of the peripheral whole blood in patients with AD; and the MMSE, MoCA, RAVLT-I, RAVLT-D, and CDR scores by using Spearman's correlation coefficient. We found positive correlations between the expression of hsa_circ_0003391 and the MMSE, MoCA, RAVLT-I, and RAVLT-D scores (Figures 4A–D), Conversely, negative correlations were found between the level of hsa_circ_0003391 and the CDR scores (Figure 4E). Furthermore, structural MRI for the hippocampus is an important neuroimaging method to make a definite diagnosis of AD cases (Kumar et al., 2017, 2019; Kumar and Reddy, 2019). The typical neuroimaging of the MRI showed that the hippocampus with the patient suffering from AD displayed an obvious atrophy compared with age-matched healthy controls (Supplementary Figure 3). We measured the hippocampus volume of the participants and made a correlation analysis. The results showed that the expression of hsa_circ_0003391 was positively correlated with the hippocampal volumes (Figures 4F–H). These results suggest that the decreased expression of hsa_circ_0003391 is significantly correlated with the clinical manifestation of AD.

### Prediction and Identification of miRNA Targets of hsa_Circ_0003391 in the Peripheral Whole Blood of Patients With AD

**Figure 5:**
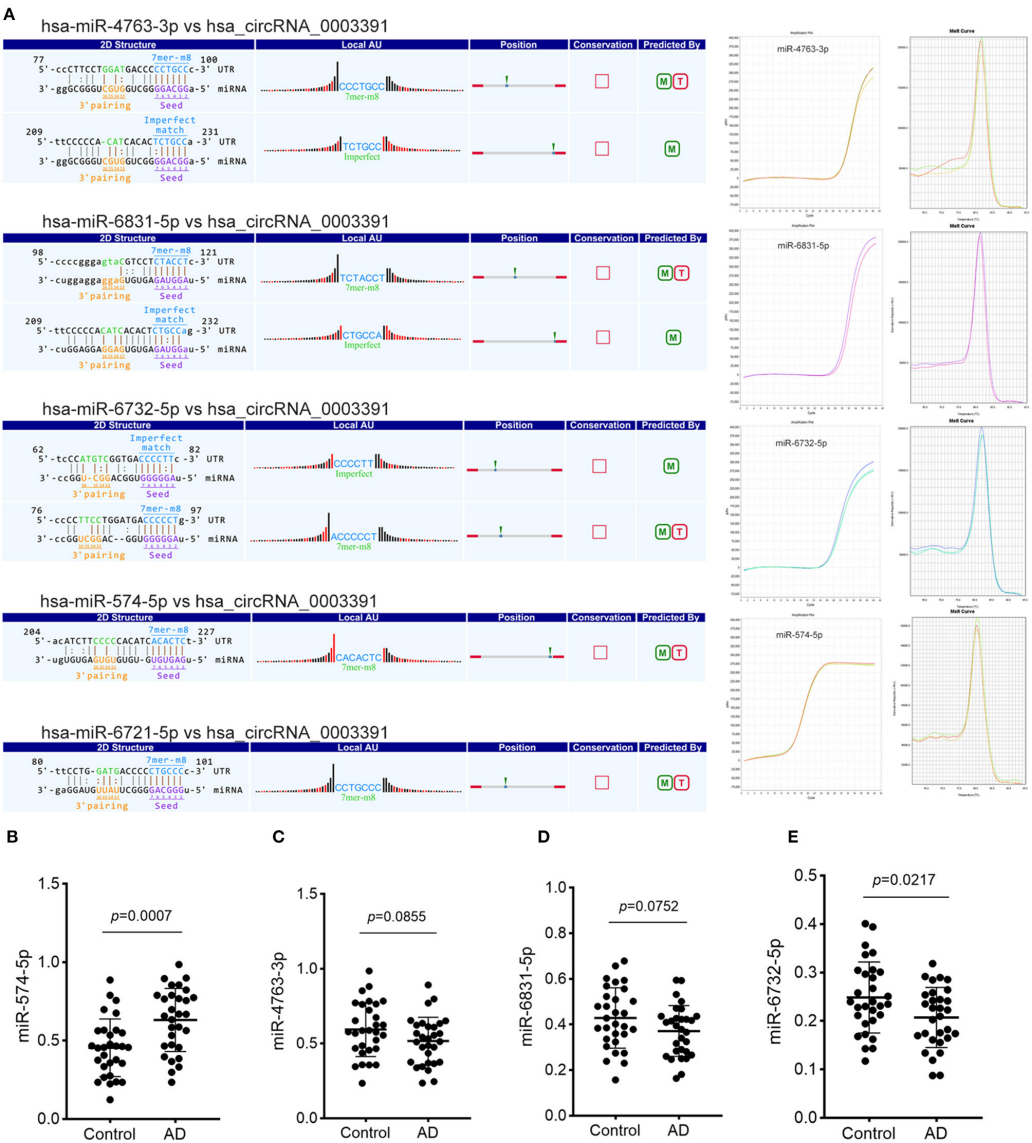
Detailed annotation of circRNA–miRNA interaction and the expression of potential miRNAs of hsa_circRNA_0003391. The circRNA–miRNA interaction was predicted using Arraystar's custom miRNA target prediction software. The amplification plots and melt curves of these miRNA targets are displayed **(A)**. The expression of potential miRNAs of hsa_circRNA_0003391 in patients with AD (*n* = 30) and age-matched controls (*n* = 30) are shown **(B–E)**. Data were analyzed using Student's *t*-test.

Although we identified the potential correlation between the expression of hsa_circ_0003391 and the clinical manifestation of AD, the function of hsa_circ_0003391 in AD remains unknown. In theory, the interactions between circRNAs and their target miRNAs should be predicted by conserved seed-matching sequences; in addition, the functions of circRNAs could be speculated according to their target miRNAs. As shown in Figure 5A, the circRNAs interacted with their five highest-ranking binding target miRNAs by the specific base pairing. Furthermore, RT-qPCR was used to detect these miRNAs in the peripheral whole blood. Melting curves with a single peak demonstrated that, of the Top-5 miRNAs, miR-4763-3p, miR-6831-5p, miR-6732-5p, and miR-574-5p were successfully detected with the exception of miR-6721-5p. CircRNAs are regarded as miRNA sponges that regulate the activity of targeted miRNAs. In order to investigate the potential target miRNAs of hsa_circ_0003391, we compared these miRNAs in the peripheral whole blood of patients with AD and their age-matched controls. The results showed that miR-574-5p had an expected elevation in the AD group and that the expression of miR-4763-3p and miR-6831-5p were not obvious in the statistical difference (Figures 5B–D). In addition, the level of miR-6732-5p in the AD group was slightly downregulated (Figure 5E). These results suggested that miR-574-5p might be a potential target of hsa_circ_0003391 in the peripheral whole blood of patients with AD.

## Discussion

The roles of circRNAs in CNS disorders are currently attracting significant attention. CircRNAs tend to accumulate during the normal process of brain aging, thus causing susceptibility to age-related neurodegenerative diseases such as AD. Recently, the significant associations between circRNA expression in the parietal cortex and AD diagnosis, clinical dementia severity, and neuropathological severity have been well-explained (Umber et al., 2019). In this study, we focused on the differentially expressed circRNAs in the peripheral blood of patients with AD, as blood is relatively easier to obtain from these patients. The close relationship between the AD and innate immunity is another reason for predicting the correlation of circRNA expression in the peripheral blood and AD.

Circular RNAs in the peripheral blood serve as potential biomarkers for the diagnosis of many diseases. For example, hsa_circ_0001275 in peripheral blood mononuclear cells has a potential ability to diagnose postmenopausal osteoporosis (Zhao et al., 2018), and hsa_circRNA_103636 is decreased in major depressive disorder patients compared with age-matched controls, and it has significantly changed after 8 weeks of antidepressant regiments (Cui et al., 2016), suggesting that hsa_circRNA_103636 could be a vital potential biomarker for the diagnosis and therapy of major depressive disorder. In our study, we used three samples from the AD plasma to perform the circRNA microarray analysis to screen the aberrant circRNAs in the initial stage, and the repeatability of the three samples was beneficial. In the validation stage, we collected 30 plasma samples to detect the aberrant circRNAs in AD. After analyzing each step, we focused on the target circRNA hsa_circ_0003391 for further study. Next, we expanded the sample size from the whole blood cells that were removed from the plasma to identify the expression of hsa_circ_0003391 in patients with AD. Compared to the age-matched control, the expression of hsa_circ_0003391 was significantly decreased in both plasma and the peripheral blood cells, with an expected higher AUC. Despite a smaller sample size in primary screening, the target circRNA hsa_circ_0003391 was successfully selected. Specificity is an important characteristic of a potential biomarker. Compared with other types of dementia, DLB, and VD, the expression of hsa_circ_0003391 in patients with AD in the peripheral blood cells displayed a specific decrease. CircRNAs are characterized by a covalently closed loop structure with neither 5 to 3 polarity nor a polyadenylated tail. The sequence of the backsplice junction demonstrates that the natural structure of hsa_circ_0003391 in the peripheral blood is circular. In addition, under different storage conditions, hsa_circ_0003391 exhibited stable characteristics in the peripheral blood of patients with AD, which is required as a potential biomarker. Furthermore, we analyzed the Spearman's correlation between hsa_circ_0003391 and the clinical manifestation of these samples. The expression of hsa_circ_0003391 was positively correlated with MMSE, MoCA, RAVLT-I, and RAVLT-D and negatively correlated with CDR. Furthermore, the levels of hsa_circ_0003391 were positively correlated with the hippocampus volumes from the MRI images, which suggests that there is a close relationship between hsa_circ_0003391 and AD. Although the correction of hsa_circ_0003391 expression with Aβ and tau should have been detected, in the actual situation, lumbar puncture is a relatively invasive procedure and is a challenge in elderly infirm individuals. Therefore, most participants were reluctant to undergo invasive examination. In our study, there were 12 participants who underwent the cerebrospinal fluid examination. According to the cognitive behavior tests and neuroimages, we deduced that the expression of hsa_circ_0003391 in the peripheral blood with AD patients has a potential ability for AD diagnosis.

The role of circRNAs in AD remains largely unknown. The most frequently reported functions of circRNAs are their role as a miRNA sponge and the formation of the circRNA–miRNA–mRNA axis (Han et al., 2017). Shobana et al. performed circRNA detection on cell-specific transcriptomic data and confirmed the potential circRNA–miRNA–mRNA regulatory networks in the posterior cingulate astrocytes of AD patients (Sekar et al., 2018). Wang et al. identified circRNA-associated-ceRNA networks in the hippocampus of AD-like rats (Wang et al., 2018). Relatively conservative in evolution, miRNA-7 is associated with circRNA (CIRS-7, also known as CDR1as) and is abundant in the human brain (Lukiw, 2013; Akhter, 2018). CiRS-7 contains multiple, tandem anti-miRNA-7 sequences that act as endogenous, anti-complementary miRNA “sponges” to adsorb and quench normal miRNA-7 functions (Lukiw, 2013; Akhter, 2018). Dysregulation of the miR7-ciRS-7 interaction has been reported in the hippocampus of patients with AD (Lukiw, 2013; Floris et al., 2017; Akhter, 2018). We also used bioinformatics methods to predict the circRNA–miRNA interaction of hsa_circ_0003391 and miRNAs, and hsa_circ_0003391 had Top-5 of the potential miRNAs. We detected that the expression of miR-574-5p had a significant elevation in the peripheral blood of AD patients. The miR-574-5p has many biological functions in oncobiology. In neuroscience field, miR-143-5p is obviously upregulated in the cerebrospinal fluid of sporadic amyotrophic lateral sclerosis compared to healthy controls (Freischmidt et al., 2013). It is worth noting that the miR-574-5p expression can be specifically repressed in the cortex and neural progenitors by amyloid precursor protein to inhibit neurogenesis (Zhang et al., 2014). In our future studies, we will explore that the potential molecular mechanism of the increased miR-574-5p in the peripheral blood of patients with AD will be regulated by hsa_circ_0003391 and will further clarify its role in the pathogenesis of AD.

In conclusion, our findings reveal that the expression of hsa_circ_0003391 is significantly decreased in the peripheral blood of patients with AD, and this decrease is correlated with the clinical features of patients with AD. Our findings provide an important potential biomarker for AD as well as a reference and inspiration for the diagnostic methodology of AD. This study also highlights a novel perspective to direct further research into AD pathogenesis, facilitating the development of novel therapies that target non-coding RNA.

## Data Availability Statement

The data can be found in the GEO - GSE161435.

## Ethics Statement

The studies involving human participants were reviewed and approved by Ethics committee of China Medical University. The patients/participants provided their written informed consent to participate in this study.

## Author Contributions

LL and KZ designed this work. LL and XC carried out the experiments and analyzed the data. KZ and Y-HC wrote and corrected the manuscript. All authors contributed to the article and approved the submitted version.

## Conflict of Interest

The authors declare that the research was conducted in the absence of any commercial or financial relationships that could be construed as a potential conflict of interest.
