# Supplementary material for: Identification of Circular RNA hsa_Circ_0003391 in Peripheral Blood Is Potentially Associated With Alzheimer's Disease
Source: Front Aging Neurosci. 2020 Dec 18;12:601965. doi: 10.3389/fnagi.2020.601965 (PMC7793744; doi:10.3389/fnagi.2020.601965)
Supplement: Supplementary file 1 [file Data_Sheet_1.pdf]

**Supplementary Figures**

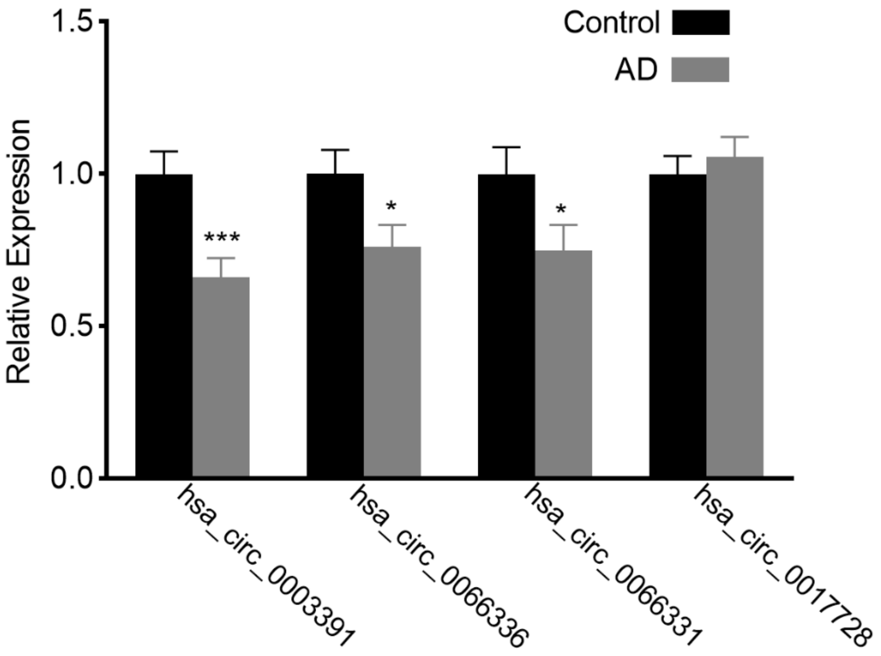


**Supplementary Figure 1**. Relative change in gene expression were determined by using a $2^{-\Delta\Delta CT}$ method. $^{*}p<0.05$, $^{***}p<0.001$

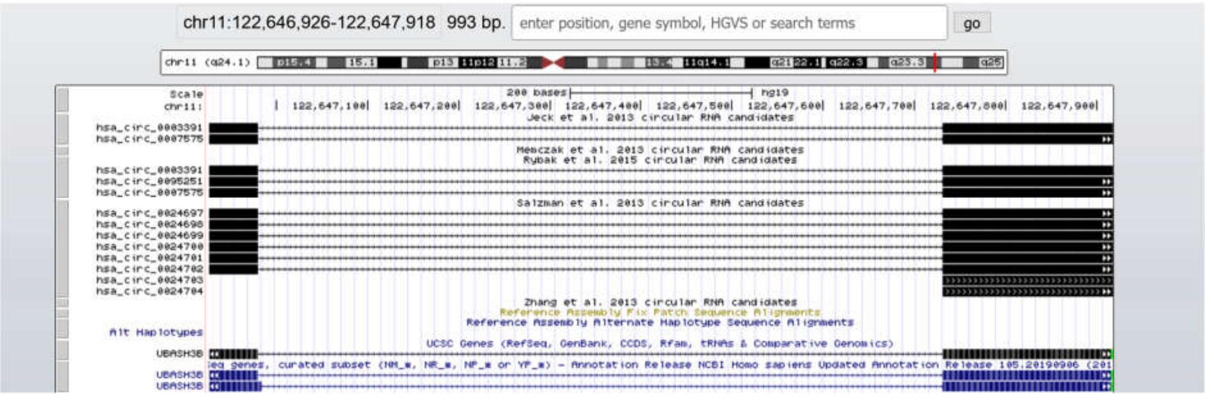


**Supplementary Figure 2**. The detail information of hsa_circ_0003391 in USPS database.

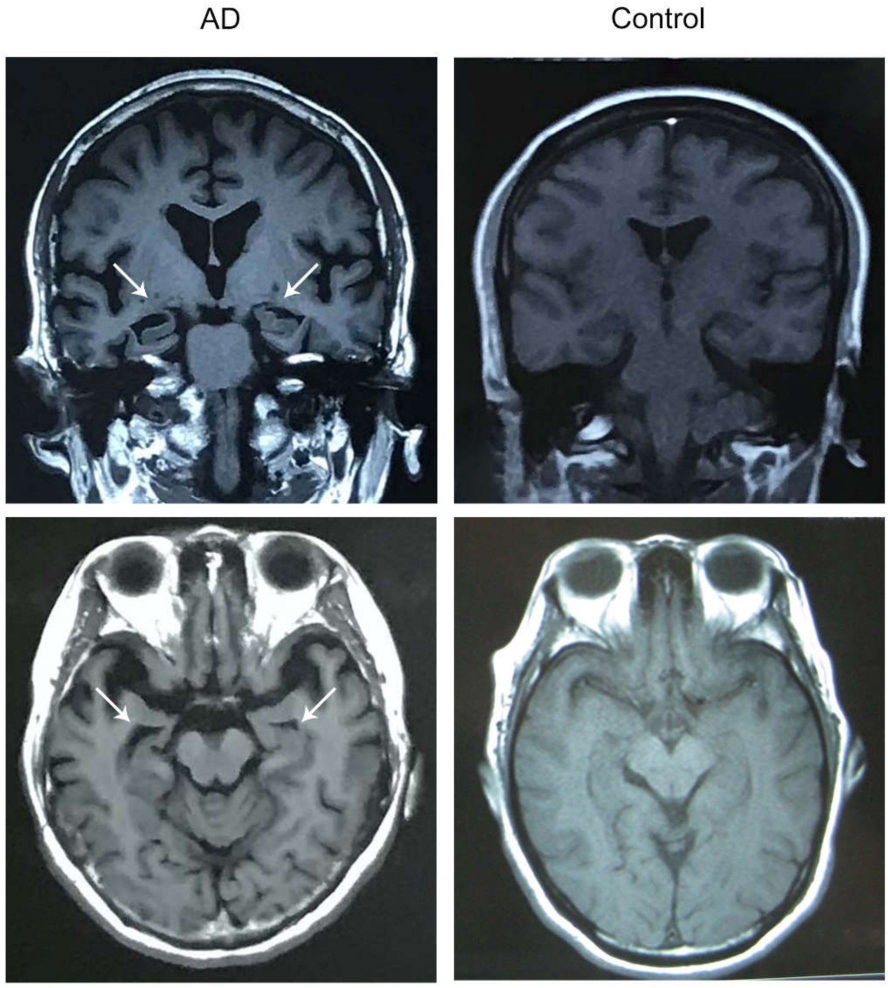


**Supplementary Figure 3**. Typical neuroimaging of the MRI showed that the hippocampus with the patient suffering from AD displayed an obvious atrophy compared with age-matched healthy controls.
